# An Interpretable Deep Learning Framework for Spatial Prediction of Soil Salinity Using Multi-Source Remote Sensing and Environmental Data: A Case Study of Cangzhou, Hebei Province, China

**DOI:** 10.3390/s26206465

**Published:** 2026-10-13

**Authors:** Mingyuan Ye, Peng Chen, Junchi Ma, Bingxia Liu, Jiahao Qi, Yuan Jing

**Affiliations:** 1 Langfang Integrated Natural Resources Survey Center, China Geological Survey, Tianjin 300301, China; yemingyuan@mail.cgs.gov.cn (M.Y.); majunchi@mail.cgs.gov.cn (J.M.); jingyuan@mail.cgs.gov.cn (Y.J.); 2 School of Water Resources and Environment, China University of Geosciences (Beijing), Beijing 100083, China; 3 Center for Agricultural Resources Research, Institute of Genetics and Developmental Biology, Chinese Academy of Sciences, Shijiazhuang 050021, China

**Keywords:** soil salinity prediction, Sentinel-2, multi-source remote sensing, interpretable deep learning, environmental covariates

## Abstract

Soil salinity poses a significant challenge to agricultural productivity and sustainable land management in coastal and semi-arid regions. Soil salinity in coastal agricultural regions is controlled by complex interactions among surface conditions, groundwater processes, soil properties, and terrain characteristics. Accurate spatial prediction requires integrating remote sensing observations with environmental information that represents these processes. This study presents a multi-source deep learning framework that integrates Sentinel-2-derived spectral indices with environmental covariates for regional soil salinity spatial prediction under the environmental conditions of the 2024 sampling period. SHapley Additive exPlanations (SHAP) is employed to quantify the contribution of environmental variables within the prediction framework and improve model interpretability. A convolutional neural network (CNN) incorporating a squeeze-and-excitation (SE) attention mechanism is developed as a nonlinear feature representation framework for integrating heterogeneous predictors, while the Crested Porcupine Optimizer (CPO) is used to optimize model hyperparameters. The proposed framework achieved the highest predictive performance among the benchmark models, with an R^2^ of 0.785, an RMSE of 0.928, and an MAE of 0.720. Integrating environmental covariates with spectral features further improves the prediction performance, increasing the R^2^ from 0.701 to 0.785. The results indicate the potential of integrating multi-source environmental information with interpretable deep learning for spatial prediction of near-surface soil salinity under similar coastal agricultural conditions.

## 1. Introduction

Soil salinity is one of the most widespread forms of global land degradation, posing substantial challenges to agricultural productivity and ecosystem sustainability [1]. Excessive accumulation of soluble salts deteriorates soil structure and reduces soil permeability while increasing soil solution osmotic pressure. This process restricts plant water uptake, induces physiological drought, and consequently reduces crop productivity, threatening regional food security and sustainable land management [2].

Cangzhou City, located in the eastern part of the North China Plain, is a typical semi-arid region characterized by water scarcity and high evaporation rates, and represents one of the major coastal soil salinity-affected areas in China [3]. The spatial distribution of soil salinity in this region is associated with complex interactions among physiographic conditions, hydrogeological settings, and human activities. Severe soil salinity has developed under these combined influences, substantially limiting agricultural productivity [4]. Therefore, accurate spatial characterization of soil salinity in this region is essential for sustainable land use and agricultural management. In coastal regions, soil salinity is governed by coupled soil–water–salt processes. Shallow saline groundwater can act as an important salt source, and upward capillary movement under strong evaporative conditions may promote salt accumulation in near-surface soils. Meanwhile, soil texture influences water retention and drainage conditions, while terrain characteristics affect local water redistribution and spatial variability of salinity. Therefore, environmental variables describing hydrological conditions, soil properties, and terrain characteristics may provide complementary information beyond surface spectral observations.

Remote sensing techniques have been widely applied for soil salinity spatial prediction, through the integration of salinity-sensitive spectral indices derived from multispectral imagery with machine learning models [5,6,7]. Conventional machine learning methods such as Random Forest (RF), Backpropagation Neural Networks (BPNN), Support Vector Machines (SVM), and Extreme Learning Machines (ELM) have been extensively used for soil salinity estimation [8,9,10,11]. For example, Chen et al. integrated Sentinel-2 imagery with spectral variables to enhance salinity signal extraction and achieved soil salinity prediction in a coastal agricultural region [12]. Yin et al. combined Sentinel-1 SAR features with Sentinel-2 spectral indices and showed that multi-source data fusion improves Random Forest-based salinity estimation performance [13]. Huang et al. used Landsat OLI data to analyze spatial patterns of soil salinity and its impacts on crop growth in irrigation areas [14]. Zhou et al. developed an improved SVM-based model for soil salinity prediction in a wetland environment, achieving improved performance under optimized settings [15]. These studies demonstrate the potential of remote sensing-based approaches for soil salinity spatial prediction. However, models relying primarily on spectral information may have limited capability in heterogeneous coastal environments because soil salinity is influenced not only by surface spectral characteristics but also by hydrological conditions, soil properties, and geographical factors [16,17,18]. Recent studies have increasingly integrated remote sensing observations with environmental covariates to improve soil salinity spatial prediction, recognizing that spectral information alone may not fully represent the environmental processes controlling soil salt accumulation. For instance, Soraya Bandak used Landsat and Sentinel-2 data combined with machine learning to analyze temporal salinity variation [19]. Tehseen Javed et al. integrated remote sensing indices with environmental factors and applied machine learning and explainable methods to analyze soil salinity drivers [20]. Showmitra Kumar Sarkar et al. combined Landsat-derived indices to map coastal soil salinity and classify salinity zones [21]. These studies demonstrate that environmental variables can provide complementary information related to soil hydrological conditions, soil properties, and landscape characteristics, which are not directly captured by satellite spectral observations.

Although remote sensing techniques provide an effective means for soil salinity spatial assessment, many existing methods still primarily rely on spectral indices and empirical relationships, which may not fully represent the hydrological and environmental processes controlling soil salinity variability [22,23,24,25]. These approaches may have limited capability when applied to complex salinity environments such as Cangzhou. First, spectral information is often not integrated with key environmental variables representing soil–water–salt processes, such as groundwater conditions, soil texture, and terrain characteristics. Second, traditional modeling approaches may have limited capability in capturing nonlinear relationships between spectral responses and environmental conditions associated with soil salinity variability. Third, model performance may vary depending on sample size, model structure, parameterization, and the ability to represent nonlinear relationships among heterogeneous predictors. Moreover, although environmental variables have increasingly been incorporated into soil salinity prediction models, their contributions within nonlinear predictive frameworks and their relationships with model outputs remain insufficiently interpreted. In spatial soil salinity prediction, spatial autocorrelation may influence model evaluation because geographically adjacent samples often share similar environmental conditions. When training and testing samples are randomly divided, spatial dependence between datasets may result in optimistic estimates of model performance. Therefore, spatial validation strategies should be considered to better evaluate model generalization capability under reduced spatial dependence.

In recent years, optimization algorithms have been increasingly applied as auxiliary strategies within deep learning frameworks to improve hyperparameter selection and model stability, including Particle Swarm Optimization (PSO) [26], Genetic Algorithms (GA) [27], and Whale Optimization Algorithm (WOA) [28]. For soil salinity prediction using field observations, appropriate model configuration is particularly important because limited sample sizes may increase sensitivity to model complexity and parameter settings. Among various optimization strategies, the Crested Porcupine Optimizer (CPO), proposed in 2024, is a metaheuristic optimization method inspired by the defensive behaviors of crested porcupines [29]. It provides an automated strategy for searching suitable hyperparameter configurations in nonlinear prediction models. In this study, CPO is not considered an independent scientific contribution, but rather a hyperparameter optimization component integrated into the deep learning framework.

In this study, an interpretable multisource deep learning framework is developed to investigate whether environmental covariates representing soil–water–salt processes can provide complementary information beyond Sentinel-2 spectral indices for near-surface soil salinity spatial prediction in Cangzhou City, China. Sentinel-2-derived spectral indices are integrated with environmental variables related to soil properties, hydrological conditions, and geographic characteristics to represent complementary information associated with soil salinity variability. First, a multisource feature integration strategy is established to evaluate the added predictive value of environmental variables beyond spectral information alone. Second, an interpretable deep learning framework based on CNN-SE-Attention is developed to model nonlinear relationships among heterogeneous predictors, while CPO is used as an auxiliary strategy for hyperparameter optimization. Finally, SHAP analysis is introduced to identify the relative contribution of environmental variables within the prediction framework and provide insights into the environmental factors associated with soil salinity variability. The objectives of this study are therefore to (1) evaluate the complementary role of environmental covariates in soil salinity spatial prediction, (2) assess the capability of an interpretable deep learning framework for integrating heterogeneous predictors, and (3) investigate the contribution of environmental variables within the prediction framework under the environmental conditions represented by the May 2024 dataset.

## 2. Materials and Methods

### 2.1. Study Area

**Figure 1:**
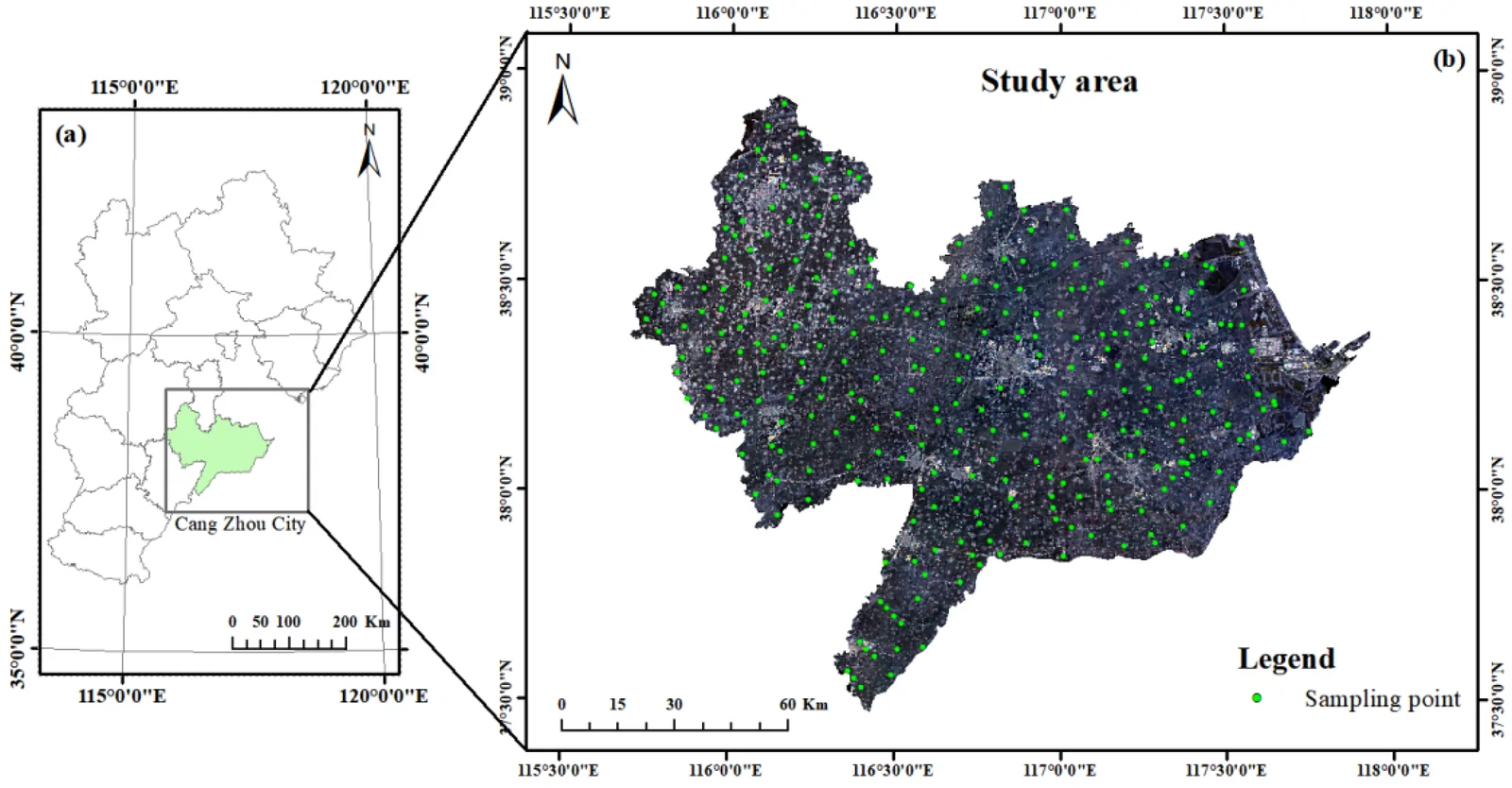
Location of the study area and distribution of soil sampling sites: (**a**) geographical location of Cangzhou City in Hebei Province, China; (**b**) Sentinel-2 imagery of the study area with sampling site distribution.

The study area is located in Cangzhou City, southeastern Hebei Province, in the eastern part of the North China Plain within the Heilonggang River Basin, adjacent to the Bohai Bay. The geographical coordinates range from 37°28′ N to 38°57′ N and 115°42′ E to 117°50′ E. The region is characterized by flat topography with a gentle slope from southwest to northeast, and elevations ranging from 2 to 17 m above sea level. Geomorphologically, the study area can be divided into three units: the western piedmont alluvial–proluvial plain of the Taihang Mountains, the central alluvial plain formed by the Haihe River system, and the eastern coastal alluvial–marine plain. The coastal plain in the eastern part is particularly low-lying and poorly drained, forming enclosed depressions where limited drainage and water redistribution may contribute to spatial variations in soil salinity [30]. The spatial distribution of the study area and sampling sites is shown in Figure 1.

The region is characterized by relatively low precipitation and high evaporation. These climatic conditions may influence soil moisture dynamics and are associated with spatial variability in surface soil salinity. Although Cangzhou is historically known as the “downstream of nine rivers”, water resources are scarce, and per capita freshwater availability is extremely limited. In addition, shallow groundwater with high salinity is widely distributed, with depths locally less than 1.0 m. Under strong evaporative conditions, salts are transported upward through capillary rise, making shallow saline groundwater a potential contributor to near-surface salt accumulation under favorable evaporation and soil salinity conditions [31]. These hydro-climatic conditions are associated with strong spatial heterogeneity of soil salinity, highlighting the need for integrating remote sensing observations with environmental information to improve spatial prediction of near-surface soil salinity.

### 2.2. Data Acquisition and Preprocessing

#### 2.2.1. Soil Total Salt Content and Environmental Factor Data Acquisition

Field sampling was conducted in May 2024 to characterize the spatial distribution of near-surface soil salinity under the environmental conditions represented by the sampling period. A total of 306 soil sampling sites were established across Cangzhou City (Figure 1) using a combination of uniform distribution and random sampling. The spatial arrangement of the sampling sites followed a checkerboard sampling layout, with a minimum spacing of more than 2 km between adjacent sites to ensure broad regional coverage while reducing spatial redundancy. The sampling sites covered the major geomorphological units of Cangzhou, including floodplain, estuarine delta, interfluvial depression, and coastal depression. Among the 306 sampling sites, 161 were located in the floodplain, 60 in the interfluvial depression, and 85 in the estuarine delta and coastal depression. Approximately 80% of the sampling sites were distributed in cropland, 15% in bare land, and 5% in salt pans. The dominant cropping system was winter wheat–summer maize. The geographic coordinates of all sampling sites were recorded using a handheld GPS with a positioning accuracy better than 3 m.

Soil samples were collected from the 0–20 cm surface layer using a portable soil auger, with one soil sample collected at each sampling site. This sampling depth was selected because it is commonly used for agricultural soil salinity assessment and represents the root-zone soil environment. The sampling strategy aimed to characterize the spatial variability of soil salinity across the study area rather than its temporal variability.

Samples were air-dried, ground, and sieved through a 2 mm mesh in the laboratory. Soil salinity was measured by preparing a soil–water mixture at a ratio of 1:5, which was stirred and left to stand for at least 2 h. Electrical conductivity (EC, dS/m) was measured using a conductivity meter and converted to soil total salt content (TSC, g/kg) using the following empirical relationship:

*TSC* = 3.2 × EC − 0.18 (1)

where TSC represents soil total salt content, and EC represents electrical conductivity measured from the 1:5 soil–water extract. The empirical relationship was established using calibration samples collected from the study region through linear regression between measured EC values and laboratory-determined soil total salt content. The EC–TSC relationship has been widely applied for soil salinity assessment in this region and was adopted to estimate soil total salt content from the measured EC values. The regression model was established using 100 representative soil samples collected from the study area.

Based on the soil–water–salt processes described above, previous studies and regional environmental characteristics, eleven environmental variables were selected to represent different categories of information associated with soil salinity spatial variability, including hydrological conditions, soil properties, topographic characteristics, and geographical location. These variables included groundwater depth, elevation, clay content, silt content, sand content, soil moisture, pH, surface structure, geomorphic type, distance to sea, and distance to rivers [32,33,34]. Groundwater depth was included because it represents hydrological conditions associated with soil salinity variability. Elevation and micro-topography characteristics were included to represent topographic variation related to soil salinity distribution. Soil texture was included because they characterize soil physical properties related to water and salt distribution. Soil moisture reflects water availability and indirectly indicates evaporation-driven salt concentration. Soil pH was included to represent soil chemical characteristics associated with salinity variability. Geomorphic type and surface structure define the spatial pattern of soil–water–salt redistribution. Distance to the sea and river were included as geographical variables representing spatial proximity to coastal and hydrological features. These variables provide complementary information related to environmental conditions associated with soil salinity distribution. Detailed information on the source, acquisition time, measurement method, and native spatial resolution of each environmental variable is summarized in Table 1. Field-measured variables were collected during the May 2024 field campaign, whereas auxiliary geospatial variables were obtained from publicly available datasets. All environmental variables were subsequently resampled to a common 10 m grid to facilitate spatial integration with Sentinel-2 imagery. This resampling was performed solely for data integration and does not imply that all variables originally possessed a native spatial resolution of 10 m.

#### 2.2.2. Sentinel-2 Satellite Image Acquisition

Sentinel-2 is a high-resolution multispectral satellite system consisting of Sentinel-2A and Sentinel-2B, with a revisit cycle of approximately 5 days. The multispectral instrument (MSI) provides 13 spectral bands ranging from visible to short-wave infrared (SWIR), with spatial resolutions of up to 10 m [35,36]. In this study, Sentinel-2 imagery acquired on 20 May, close to the field sampling date, was selected to represent the surface environmental conditions during the sampling period. The selected imagery represents the spatial condition during May 2024 and does not represent multi-seasonal or interannual salinity dynamics. Images with cloud cover less than 10% were initially screened, and cloud and cloud-shadow pixels were removed using the Sentinel-2 Scene Classification Layer (SCL) in Google Earth Engine (Level-2A). All Sentinel-2 data were acquired and preprocessed using the Google Earth Engine (GEE) platform, including image filtering, cloud masking, and spectral band extraction. The 20 m spectral bands were resampled to a 10 m spatial grid to match the spatial resolution of Sentinel-2 visible and near-infrared bands during feature extraction. The resampling method was nearest-neighbor. A single Sentinel-2 image was used rather than a temporal composite because the objective was to match the satellite observations with the field sampling period. Under vegetated conditions, the spectral response is influenced not only by soil background but also by canopy characteristics and vegetation responses to salinity stress. The spatial distribution of imagery and sampling points is shown in Figure 1.

It should be noted that Sentinel-2 spectral information represents integrated surface responses related to soil conditions, vegetation status, and surface moisture. Therefore, the spectral variables were used as predictive indicators associated with near-surface soil salinity rather than direct measurements of soil salt content.

#### 2.2.3. Remote Sensing Spectral Index Calculation

Remote sensing spectral indices provide surface information associated with soil conditions, vegetation responses, and moisture variations related to soil salinity spatial variability. Based on previous studies, several representative indices associated with soil salinity were selected (Table 2). Reflectance values from visible, red-edge, near-infrared (NIR), and short-wave infrared (SWIR) bands were extracted for each sampling site using ArcGIS 10.7. The spectral indices were then calculated and assigned to each sampling point based on its geographic coordinates. These spectral indices were used as one category of predictive variables representing surface spectral information associated with near-surface soil salinity variability. In addition, environmental variables derived from field measurements and auxiliary geospatial datasets were also incorporated as complementary input features.

It should be noted that the relationship between Sentinel-2 spectral responses and soil salinity is influenced by surface conditions. Under bare-soil conditions, spectral variations may be related to salt accumulation, soil moisture, and soil background characteristics. In vegetated areas, however, satellite reflectance represents a mixed signal affected by canopy structure, chlorophyll content, vegetation coverage, and plant responses to salinity stress. Therefore, the spectral indices used in this study were interpreted as indirect indicators associated with soil salinity rather than direct measurements of soil salt content.

The Sentinel-2 bands used in this study include B2 (Blue), B3 (Green), B4 (Red), B5 (Red-edge 1, RE1), B6 (Red-edge 2, RE2), B7 (Red-edge 3, RE3), B8 (Near-Infrared, NIR), B8A (Narrow Near-Infrared), B11 (Short-Wave Infrared 1, SWIR1), and B12 (Short-Wave Infrared 2, SWIR2). These bands were selected because they have been widely used to characterize soil surface conditions, vegetation status, and moisture-related variations in previous soil salinity studies.

### 2.3. Deep Learning Model

#### 2.3.1. SHAP-Based Feature Contribution Analysis

SHapley Additive exPlanations (SHAP) is an interpretable machine learning framework derived from cooperative game theory that quantifies the contribution of each input feature to the model prediction [42,43]. Compared with conventional correlation-based feature importance measures, SHAP provides a theoretically consistent and model-independent approach for evaluating the contribution of input variables by estimating their marginal contributions across possible feature combinations. It should be noted that environmental variables may exhibit correlations due to their shared geographical and hydrological characteristics. Therefore, SHAP values in this study are interpreted as conditional contributions within the trained model rather than independent effects or causal relationships.

Considering that the proposed framework involves a nonlinear deep learning model, KernelSHAP, a model-agnostic SHAP implementation, was adopted because it can estimate feature contributions without requiring access to the internal structure of the deep learning model. KernelSHAP approximates Shapley values through weighted linear regression and is applicable to complex nonlinear models without requiring access to their internal structure. This makes it particularly suitable for interpreting deep learning models in remote sensing applications. SHAP decomposes the model output as:

(2) $$\phi_{i}\left(v\right)=\sum_{S\subseteq N\backslash \{i\}}\frac{\left|S\right|!\left(n-\left|S\right|-1\right)!}{n!}\left[v\left(S\cup \{i\}\right)-v\left(S\right)\right]$$

where ϕ_i_(v) represents the contribution of feature *i*, N is the full feature set, S is a subset of features, |S| and n represent the size of the subset and the total number of features, respectively, while *v* denotes the model prediction corresponding to a given subset. The weight $\frac{\left|S\right|!\left(n-\left|S\right|-1\right)!}{n!}$ corresponds to the probability that, in a random permutation, all features in subset S appear before feature *i*. The overall Shapley value is thus defined as the weighted average of all marginal contributions [v(S ∪ {i}) − v(S)] across all possible subsets. In this study, Shapley values were used to quantify the relative contribution of environmental variables within the prediction framework. They were not interpreted as independent causal effects of environmental factors on soil salinity. Specifically, the contribution assigned to feature *i* is equal to the average marginal gain that this feature brings to the prediction across all possible feature coalitions. This framework provides a theoretically consistent and model-agnostic approach for quantifying feature importance, enabling interpretable analysis of complex machine learning models in remote sensing applications.

#### 2.3.2. CPO-SE-Attention-CNN Model

**Figure 2:**
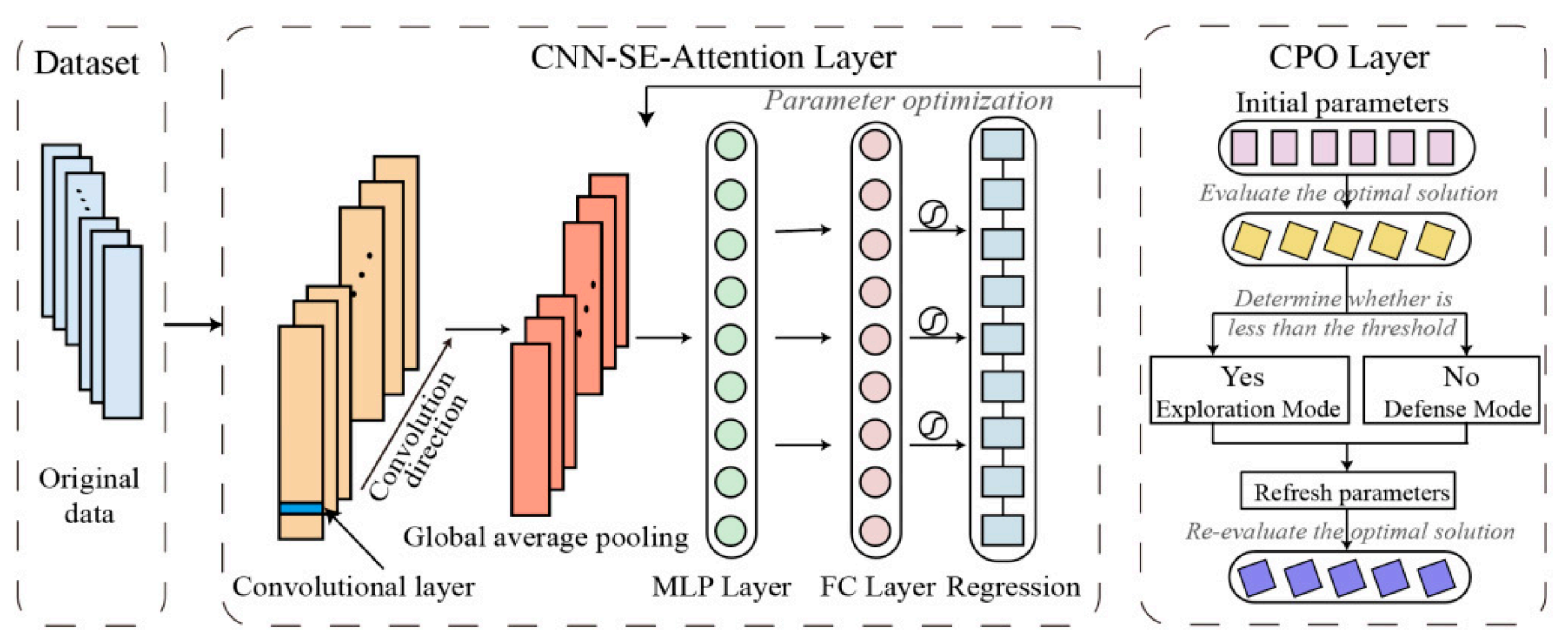
Schematic diagram of the CPO-CNN-SE-Attention algorithm.

The proposed deep learning framework is designed for nonlinear regression-based spatial prediction of soil salinity using multisource environmental information. It integrates CNN-based feature representation, SE attention-based feature recalibration, and CPO-based hyperparameter optimization. It integrates a Convolutional Neural Network (CNN) for feature extraction and a Squeeze-and-Excitation (SE) attention mechanism for adaptive channel-wise feature recalibration. The CNN component serves as a nonlinear feature representation module for extracting hierarchical interactions among multisource predictors, including environmental variables and remote sensing-derived spectral indices. The SE module is embedded within the convolutional blocks to model interdependencies among feature channels. It adaptively enhances informative features relevant to soil salinity while suppressing redundant or irrelevant information, thereby improving feature representation for regression tasks. After feature extraction and recalibration, the resulting feature maps are flattened and passed through fully connected layers to generate continuous soil salinity predictions. In this framework, CNN, SE attention, and CPO serve complementary functions. CNN extracts nonlinear feature representations, SE attention improves feature weighting, and CPO optimizes hyperparameters. Their combination aims to improve predictive performance and model stability rather than represent three independent scientific contributions. To evaluate whether convolutional representation provides additional benefits for structured tabular predictors, a multilayer perceptron (MLP) model was included as an additional benchmark. The detailed flowchart can be seen in Figure 2.

The Crested Porcupine Optimizer (CPO) is a metaheuristic optimization algorithm proposed in 2024, inspired by the defensive behaviors of crested porcupines [29]. It treats each candidate solution as a porcupine in the search space and updates the population through a dynamic balance between exploration and exploitation using a phase-switching mechanism [44,45]. At each iteration, a random number is compared with a switching parameter R to determine the search phase. If rand < R, the population enters a defensive phase; otherwise, it enters a foraging phase. Four behavioral strategies are used to update solutions.

The first strategy is the visual intimidation mechanism, which drives the solution toward the current best position to enhance local exploitation:

X_new_ = X_current_ + α ∗ δ ∗ (X_best_ − X_current_) (3)

where X_current_ and X_best_ denote the current and global best solutions, respectively, α is a scaling factor, and δ is a random vector in the interval [0, 1].

The second strategy is the acoustic warning mechanism, which introduces a time-dependent step size to balance exploration and exploitation:

X_new_ = X_current_ + (2 ∗ rand − 1) ∗ (X_best_ − X_current_)/t (4)

where rand ∈ [0, 1] and t denotes the current iteration number.

The third strategy is the olfactory attack mechanism, which incorporates Levy flight to enhance global exploration capability:

X_new_ = X_current_ + L ∗ D ∗ (X_best_ − X_current_) (5)

where L follows a Levy distribution, and D is a random direction vector.

The fourth strategy is the physical attack mechanism, which combines directed search and random differential perturbation:

X_new_ = X_current_ + K ∗ (X_best_ − X_current_) + h ∗ (X_r1_ − X_r2_) (6)

where K and h are scaling factors, and X_r1_ and X_r2_ are two randomly selected individuals from the population.

In this framework, each porcupine position encodes a set of hyperparameters of the CNN-SE-Attention model (e.g., learning rate and number of convolutional filters) [46]. The validation RMSE was used as the fitness function, and lower RMSE values indicated better hyperparameter configurations. Through iterative updates, CPO automatically searches for the optimal hyperparameter configuration, replacing manual tuning and identifying the optimal hyperparameter configuration.

The optimization process consisted of four steps: (1) initializing the CPO population; (2) encoding each individual as a CNN-SE-Attention hyperparameter configuration; (3) calculating validation RMSE as the fitness function; and (4) updating candidate solutions until the maximum iteration number was reached. The optimal hyperparameter combination was then used for final model training.

Overall, the proposed methodology integrates multi-source remote sensing data and field-measured environmental variables within a unified deep learning framework for soil salinity spatial prediction in the Cangzhou region. Sentinel-2-derived spectral indices and environmental variables are jointly used as input features to represent complementary spectral and environmental information associated with soil salinity variability. To improve model interpretability, SHapley Additive exPlanations (SHAP) is employed to quantify the contribution of environmental variables to model predictions. The selected features are then combined with spectral indices to construct the final input dataset. A hybrid deep learning model (CPO-CNN-SE-Attention) is developed to capture complex nonlinear relationships between soil salinity observations and multisource predictor variables, where the CNN-SE architecture is responsible for feature extraction and adaptive channel recalibration. In addition, the Crested Porcupine Optimizer (CPO) is introduced to automatically optimize model hyperparameters, enhancing convergence stability and prediction accuracy. The performance of the proposed model is evaluated against multiple benchmark algorithms, and its effectiveness is further validated through comparison with spectral-only results. This integrated framework provides a data-driven and interpretable approach for soil salinity spatial prediction in heterogeneous coastal environments and supports high-resolution salinity mapping under the environmental conditions represented by the study dataset. Ablation experiments were conducted to evaluate the individual contribution of the SE attention module and CPO optimization. CNN, CNN-SE, and CPO-CNN-SE models were compared using identical data partitions and repeated runs.

#### 2.3.3. Performance Evaluation Metrics

Model performance is evaluated using root mean square error (RMSE), mean absolute error (MAE), and coefficient of determination (R^2^):

$$\mathrm{RMSE}=\sqrt{\frac{1}{n}\sum_{1}^{n}{(\overset{ˇ}{y}}^{2}-y^{2})};$$

(7) $$\;\mathrm{MAE}=\frac{1}{n}\sum_{1}^{n}\left|\overset{ˇ}{y}-y\right|;$$

$$\;R^{2}=1-\frac{\sum_{1}^{n}{\left(\overset{ˇ}{y}-y\right)}^{2}}{\sum_{1}^{n}{\left(\bar{y}-y\right)}^{2}};$$

where y is observed soil salinity, $\overset{ˇ}{y}$ is the predicted value, and $\bar{y}$ is the mean observed value.

### 2.4. Soil Salinity Spatial Prediction Workflow

**Figure 3:**
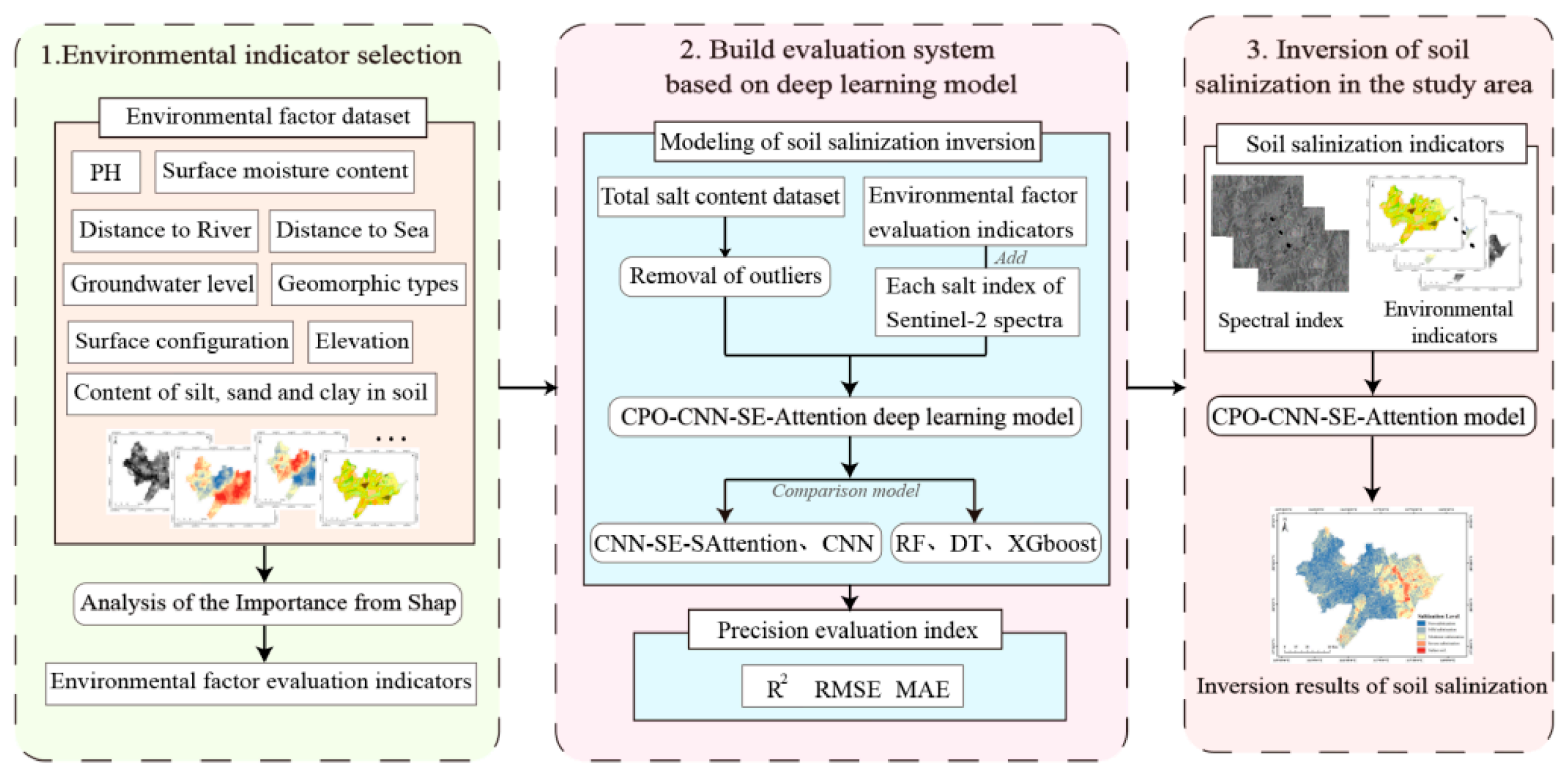
Technical workflow of soil salinity spatial prediction.

The workflow consists of five steps. First, data collection was conducted, including 306 field samples and 11 environmental indicators such as soil salinity, groundwater depth, elevation, geomorphic type, distance to sea, and soil texture. Second, the dataset was divided into training and testing subsets. SHAP-based feature contribution analysis was conducted using the training dataset only to identify important environmental variables and reduce potential information leakage during feature selection. Third, the proposed CPO-CNN-SE-Attention model was trained and compared with benchmark models including Random Forest and CNN. In addition, spatial block cross-validation was conducted to evaluate model generalization under reduced spatial dependence and avoid overly optimistic performance estimates caused by spatial autocorrelation. Fourth, model performance was evaluated using RMSE, MAE, and R^2^. To reduce uncertainty caused by random initialization and data partitioning, each experiment was repeated ten times. Mean values and standard deviations of evaluation metrics were reported. Finally, the trained model was applied to generate a spatial distribution map of near-surface soil salinity across the Cangzhou region, followed by comparative analysis between multi-source and spectral-only models. The overall workflow is illustrated in Figure 3.

## 3. Results and Analysis

### 3.1. Soil Salinity Statistical Analysis

A total of 306 soil samples were collected in this study, and 304 valid samples were retained after removing incomplete or abnormal records. These samples provided broad spatial coverage across Cangzhou and were used to characterize the spatial variability of near-surface soil salinity under the environmental conditions represented by the May 2024 sampling period. The statistical characteristics of soil total salt content were analyzed based on the 304 samples. The mean soil salinity was 2.01 g/kg, with a minimum value of 0.141 g/kg, a maximum value of 20.9 g/kg, and a standard deviation of 2.68 g/kg, indicating strong spatial variability across the study area.

According to the soil salinity classification standard [46], the samples were divided into five salinity levels. The results show that non-saline and mildly saline soils dominate the study area, while higher salinity levels are mainly distributed in localized regions. Specifically, 151 samples were classified as non-saline, 68 as mildly saline, 54 as moderately saline, 13 as severely saline, and 18 as saline soils. These results indicate a clear spatial heterogeneity of soil salinity in the study area (Table 3).

The distribution of samples among salinity categories was uneven. Non-saline and mildly saline soils accounted for the majority of observations, whereas only 18 samples were classified as saline soils (>6 g/kg). Therefore, although the dataset provided broad spatial coverage, the model may have greater uncertainty when predicting extreme salinity conditions due to limited observations in this range.

### 3.2. Construction of the Multi-Source Environmental Variable Dataset

The spatial distribution of soil salinity in Cangzhou is associated with geographical gradients, hydrological conditions, soil properties, and geomorphological characteristics that jointly influence soil–water–salt redistribution. Based on soil–water–salt processes described above, previous studies, regional environmental characteristics, and data availability, eleven environmental variables were initially considered, including groundwater depth, elevation, soil moisture content, clay content, silt content, sand content, pH, surface structure, geomorphic type, distance to the sea, and distance to rivers.

The environmental variables obtained from field measurements and auxiliary geospatial datasets were transformed into continuous spatial datasets prior to model construction. Point-based measurements, including groundwater depth, soil moisture, clay content, silt content, sand content, and pH, were spatially interpolated into continuous raster layers using ordinary kriging, while distance-to-sea and distance-to-river variables were generated using Euclidean distance analysis in ArcGIS. Elevation and geomorphological information were obtained from auxiliary geospatial datasets. All environmental variable layers were resampled to a 10 m grid for spatial alignment with Sentinel-2 imagery during feature extraction. This resampling was performed only for data integration and does not imply that all variables originally contain information at a native 10 m spatial resolution. This resampling was performed for spatial alignment and does not imply that all environmental variables originally contain information at a native 10 m spatial scale.

To interpret the contribution of environmental variables within the prediction framework, SHapley Additive exPlanations (SHAP) were employed for feature importance analysis. Since the proposed soil salinity spatial prediction model is a convolutional neural network, the KernelSHAP algorithm, a model-agnostic SHAP implementation, was adopted to estimate the contribution of each environmental variable to the model prediction. The global importance of each variable was calculated as the mean absolute SHAP value across all samples. Environmental variables were ranked according to their mean absolute SHAP values. Therefore, SHAP results in this study should be interpreted as model-dependent associations between environmental variables and predicted soil salinity rather than direct evidence of physical causality.

**Figure 4:**
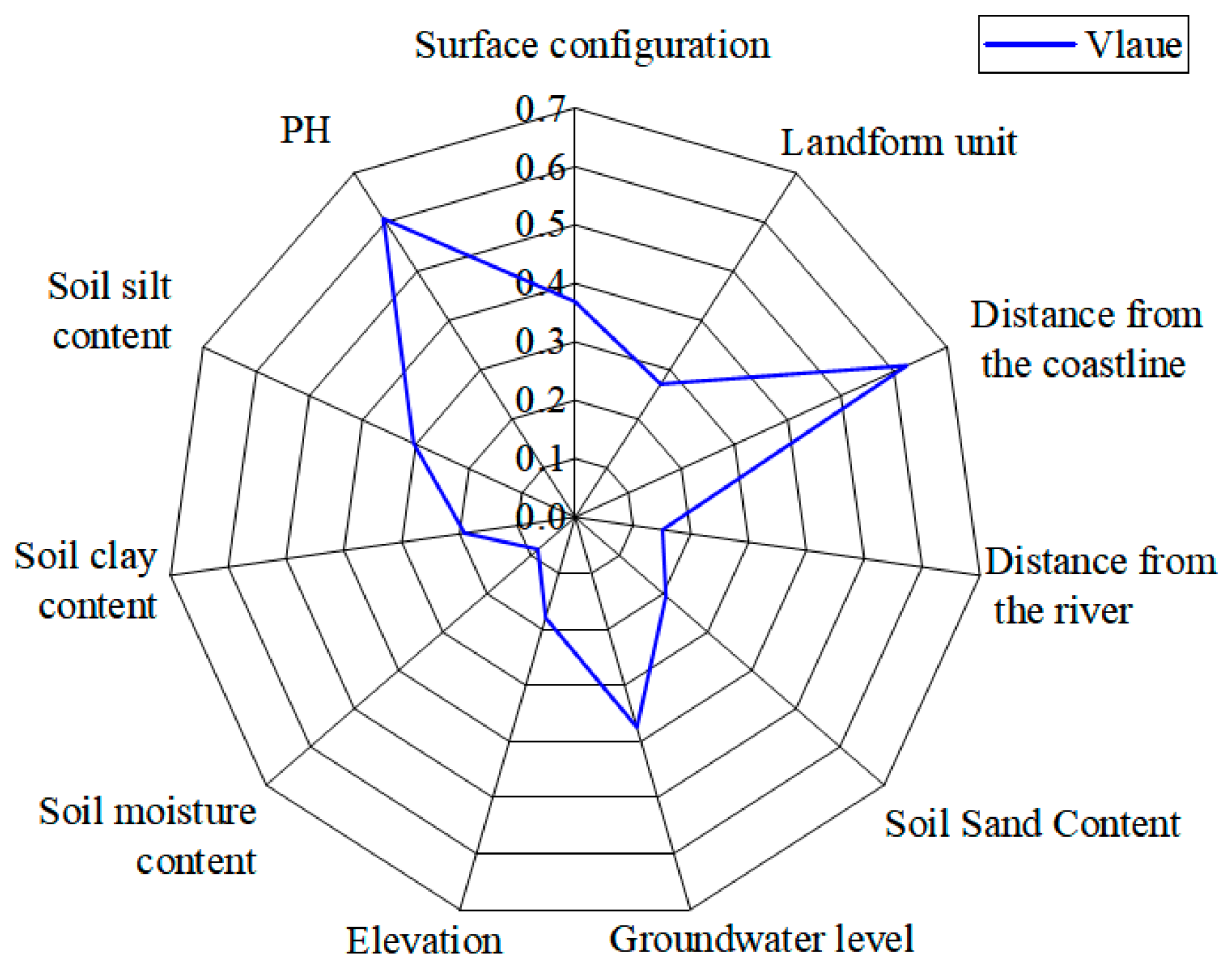
Pie chart of SHAP-derived feature importance for environmental variables related to soil salinity prediction.

**Figure 5:**
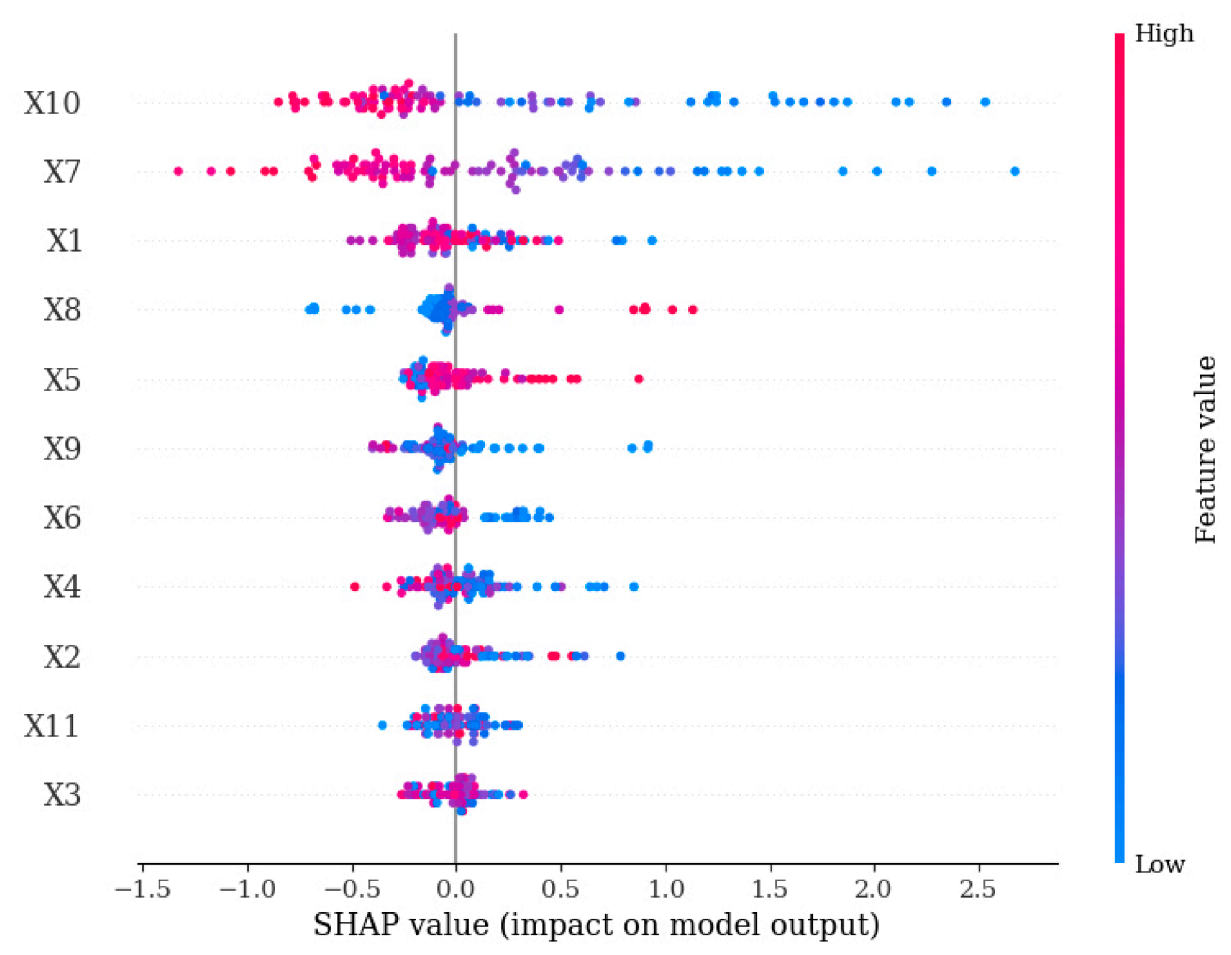
SHAP-based importance ranking of environmental variables associated with soil salinity. X1–X11 denote groundwater depth, elevation, soil moisture content, clay content, silt content, sand content, pH, surface structure, geomorphic type, distance to the sea, and distance to rivers, respectively.

The SHAP results (Figure 4 and Figure 5) indicate that distance to the sea, pH, groundwater depth, surface structure, and silt content showed relatively higher contributions to model predictions.

Among these variables, distance to the sea exhibited the highest SHAP contribution value (0.6242). This variable represents the spatial relationship between sampling locations and coastal areas, and its higher contribution suggests that coastal proximity provides important predictive information for soil salinity estimation. Soil pH ranked second, with an importance value of 0.6072. As an indicator of soil chemical conditions, Soil pH represents soil chemical characteristics associated with variations in soil salinity. Groundwater depth exhibited an importance value of 0.3752 and represents hydrological conditions associated with soil salinity variability. Shallower groundwater levels may provide favorable conditions for upward salt migration under appropriate evaporation and soil hydraulic conditions; however, groundwater depth alone does not directly represent groundwater salt flux. Surface structure showed an importance value of 0.3686. Surface structure showed a relatively high contribution, indicating its predictive relevance within the trained model. Soil silt content ranked fifth, with an importance value of 0.3039. Silt content represents soil physical properties that may be associated with soil salinity variability. It should be noted that SHAP analysis quantifies the contribution of variables within the prediction model and does not independently establish causal relationships. Therefore, the identified variables should be interpreted as important predictive factors under the environmental conditions represented by the dataset.

Collectively, these five variables represent different categories of environmental information, including coastal gradients, chemical environment, hydrodynamic conditions, topographic controls, and soil physical properties. Their combined effects play a critical role in shaping the spatial distribution of soil salinity in the study area.

These variables provide complementary environmental information for soil salinity prediction. To further improve model input representation, Sentinel-2-derived spectral indices were also extracted. A total of eight salinity-sensitive indices (NDVI, SRSI, and SI–SI6) were calculated and integrated with environmental variables to construct a multi-source feature dataset for soil salinity spatial prediction.

### 3.3. Construction of Soil Salinity Spatial Prediction Models

Based on SHAP-based feature contribution analysis conducted using the training dataset, the selected environmental variables and eight Sentinel-2-derived spectral indices were used as predictor variables, while the measured soil total salt content was used as the response variable. The dataset was randomly divided into training (70%) and testing (30%) subsets for model comparison, while spatial block cross-validation was additionally conducted to evaluate model generalization under reduced spatial dependence. Considering that random partitioning may introduce spatial dependence between training and testing samples, spatial block cross-validation was performed as an additional evaluation strategy.

**Figure 6:**
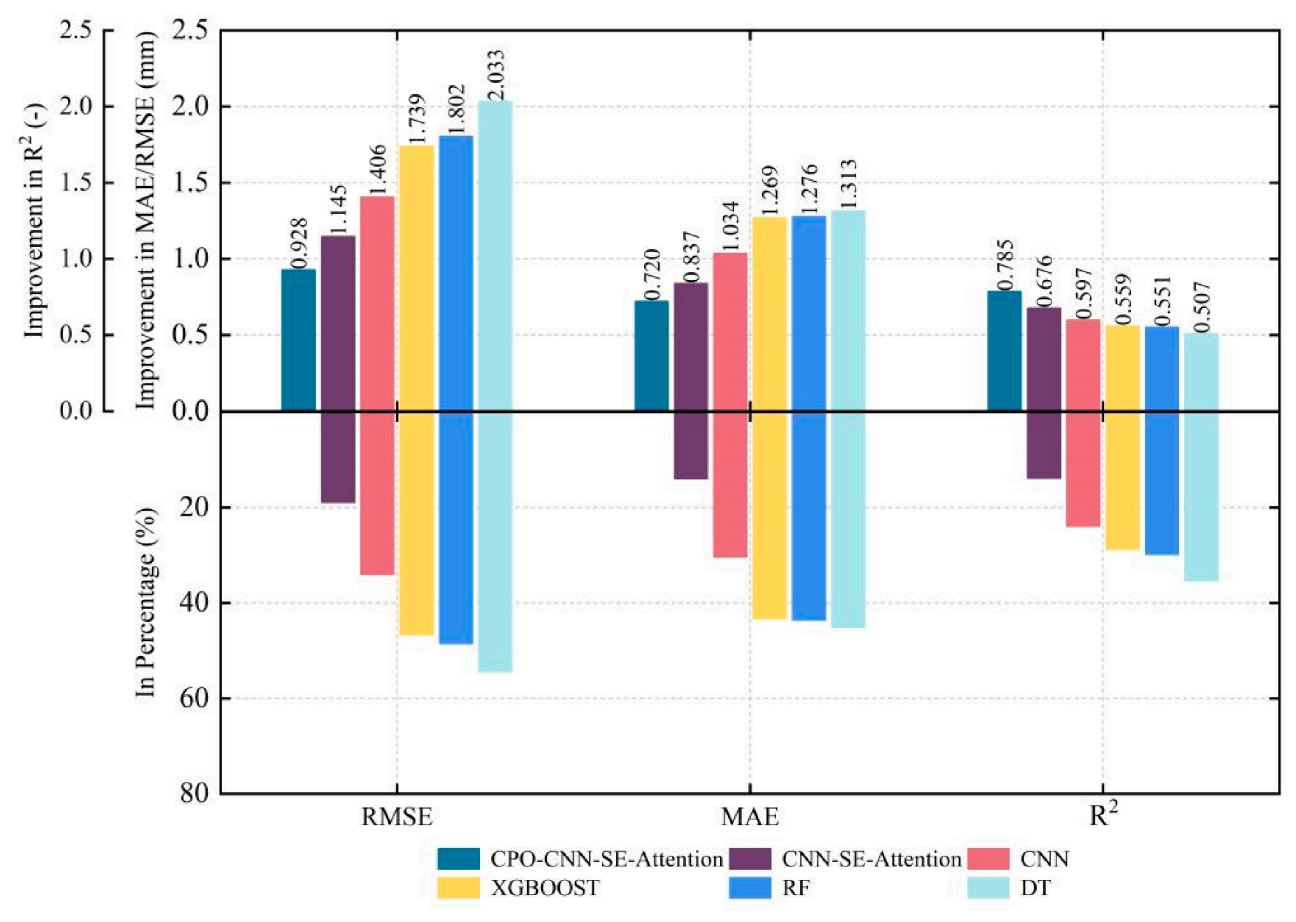
Performance comparison of the proposed CPO-CNN-SE-Attention model and benchmark models on the testing dataset based on RMSE, MAE, and R^2^.

**Figure 7:**
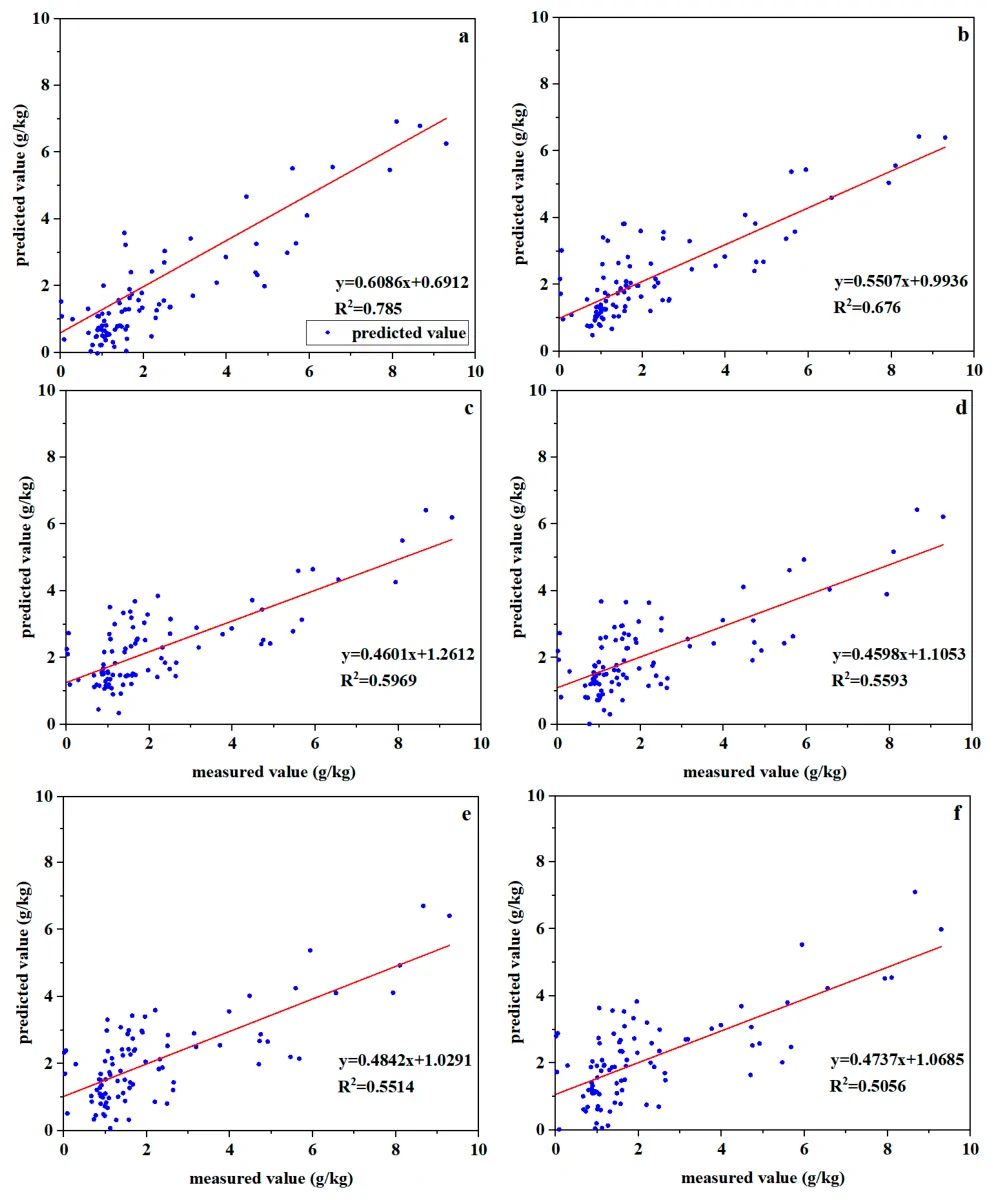
Comparison of soil salinity spatial prediction results obtained using different models on the testing dataset: (**a**) CPO-CNN-SE-Attention; (**b**) CNN-SE-Attention; (**c**) CNN; (**d**) XGBoost; (**e**) Random Forest; and (**f**) Decision Tree.

A hybrid deep learning framework incorporating CNN-based feature representation, SE attention, and CPO-based hyperparameter optimization was developed for soil salinity spatial prediction. The CNN component was employed to extract nonlinear features from multi-source inputs, whereas the SE attention mechanism was incorporated to adaptively recalibrate channel-wise feature responses. The Crested Porcupine Optimizer (CPO) was adopted to optimize model hyperparameters, thereby improving convergence stability and prediction accuracy. The principal parameter settings of the proposed model are summarized in Table 4. In this study, the CPO algorithm was employed to optimize the key hyperparameters of the CNN-SE-Attention model. Each candidate solution in the CPO population represented a specific hyperparameter configuration, including the learning rate, number of convolutional filters, dropout rate, and the number of neurons in the fully connected layers. To ensure a fair comparison, all benchmark machine learning models were optimized using grid search based on the training dataset. For XGBoost, the optimized parameters included the learning rate, maximum tree depth, and number of estimators. The optimal configuration was obtained as learning rate = 0.01, maximum depth = 3, and number of estimators = 200. For Random Forest, the main optimized parameters included the number of trees, maximum depth, and minimum samples required for node splitting, with the optimal values of 300, 5, and 3, respectively. For Decision Tree, the optimized parameters included maximum depth, minimum samples split, and minimum samples leaf, with optimal values of 12, 8, and 5, respectively. These optimized configurations were used for model comparison with the proposed CNN framework. The search ranges of these hyperparameters were predefined according to the model architecture and empirical settings (Table 4 and Table 5). During the optimization process, the validation RMSE was used as the fitness function, and the objective of CPO was to identify the hyperparameter combination that minimized prediction error. The candidate solutions were iteratively updated through the exploration and exploitation mechanisms of CPO until the maximum number of iterations was reached. The optimal hyperparameter configuration obtained from CPO was subsequently used to train the final CNN-SE-Attention model for soil salinity spatial prediction.The detailed results are shown in Figure 6 and Figure 7.

To evaluate the robustness of different prediction models, each model was independently trained and evaluated ten times under the same training-testing partition and parameter optimization strategy. The mean values and standard deviations of the evaluation metrics were calculated to account for variations caused by random initialization.

The proposed CPO-CNN-SE-Attention model achieved the highest predictive performance among all evaluated models, with an R^2^ of 0.785 ± 0.022, RMSE of 0.928, and MAE of 0.720. Compared with the baseline CNN model (R^2^ = 0.597 ± 0.033, RMSE = 1.406, MAE = 1.034), incorporating the SE attention mechanism improved the prediction performance (R^2^ = 0.676 ± 0.027, RMSE = 1.145, MAE = 0.837). Further optimization using the CPO algorithm increased the prediction accuracy to R^2^ = 0.785 ± 0.022 while further reducing the RMSE to 0.928.

To further evaluate the suitability of the convolution-based architecture for structured tabular predictors, a multilayer perceptron (MLP) model was additionally implemented. The MLP achieved an R^2^ of 0.584 ± 0.026, which was slightly lower than that of the baseline CNN (R^2^ = 0.597 ± 0.033), suggesting that the convolution-based feature representation provided a modest improvement over a conventional fully connected neural network under the experimental conditions. Among the conventional machine learning models, XGBoost (R^2^ = 0.559 ± 0.031), Random Forest (R^2^ = 0.551 ± 0.032), and Decision Tree (R^2^ = 0.506 ± 0.032) showed progressively lower prediction accuracy. All benchmark models were optimized using their respective hyperparameter tuning strategies before comparison to ensure a fair evaluation.

Overall, these results suggest that the SE attention mechanism and CPO-based hyperparameter optimization contributed to improved predictive performance within the proposed framework, while the CNN backbone provided more effective nonlinear feature representation than the evaluated conventional machine learning models and the baseline MLP under the conditions of this study. To further evaluate the contribution of environmental variables, two experimental scenarios were designed: (1) using only Sentinel-2-derived spectral indices and (2) combining spectral indices with the selected environmental variables. The same CPO-CNN-SE-Attention architecture was applied in both experiments. This experiment was designed to isolate the contribution of environmental covariates beyond spectral information. To assess model stability, each experiment was repeated ten times, and the mean values and standard deviations of R^2^, RMSE, and MAE were calculated to evaluate model stability.

**Figure 8:**
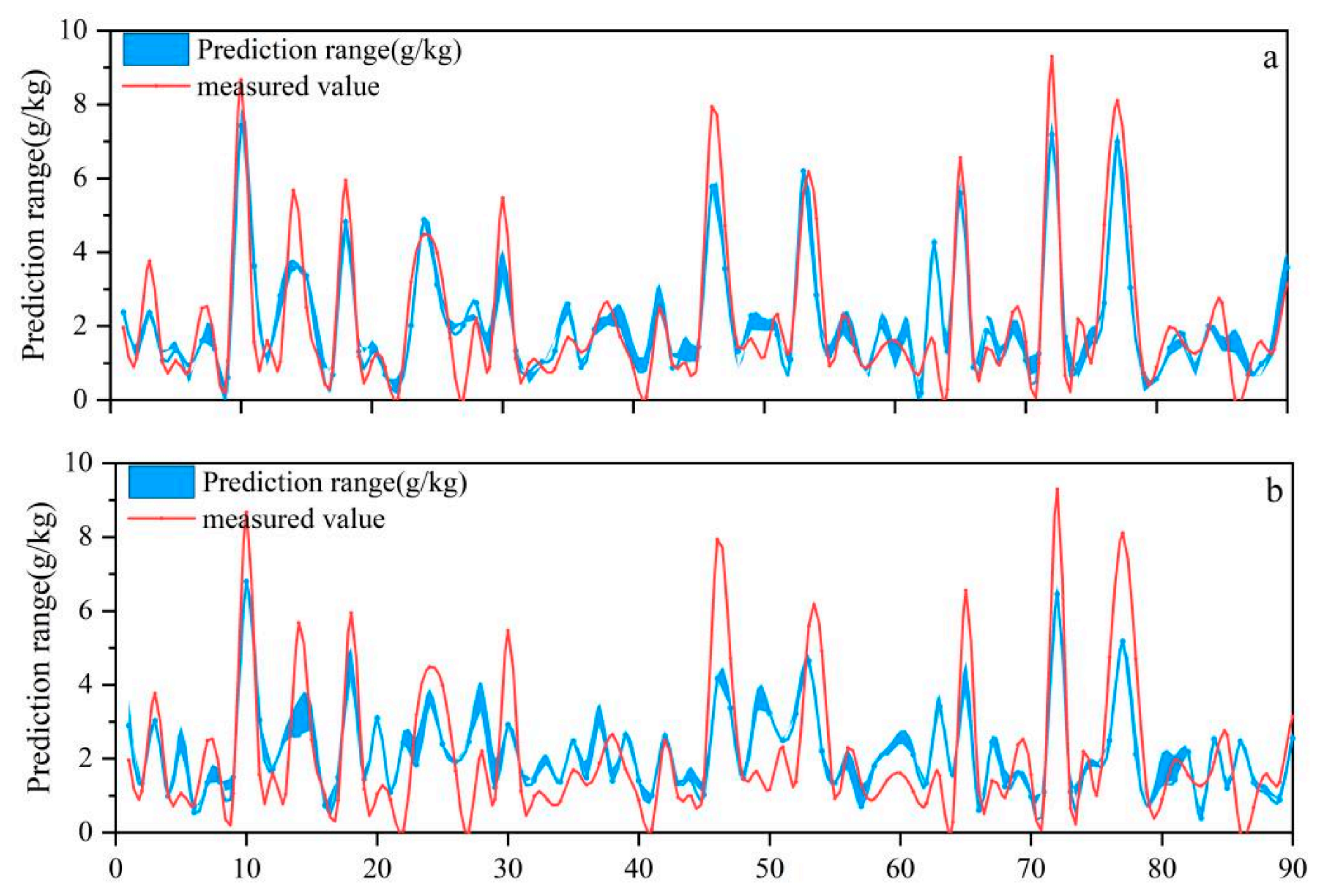
Comparison of spatial prediction results with and without environmental variables: (**a**) with environmental variables; (**b**) only spectral indices.

The statistical results from ten independent model runs indicate that incorporating environmental variables improves model performance. Specifically, the mean R^2^ increased from 0.701 to 0.785, whereas the mean RMSE decreased from 1.084 to 0.928. The proposed framework achieved an average R^2^ of 0.785 ± 0.022, an RMSE of 0.928 ± 0.034, and an MAE of 0.720 ± 0.019 across repeated experiments, indicating relatively stable predictive performance. These findings indicate that environmental variables provide complementary predictive information beyond spectral responses alone, particularly by representing variations in hydrological conditions, soil properties, and geographical characteristics. The detailed results are shown in Figure 8.

Although the maximum observed soil salinity reached 20.9 g/kg, only 18 samples belonged to the saline-soil category (>6 g/kg). Consequently, the evaluation metrics were primarily influenced by non-saline and mildly saline samples. The prediction performance for highly saline soils is further discussed in Section 4.

### 3.4. Predicted Spatial Distribution of Soil Salinity Mapping of the Study Area

**Figure 9:**
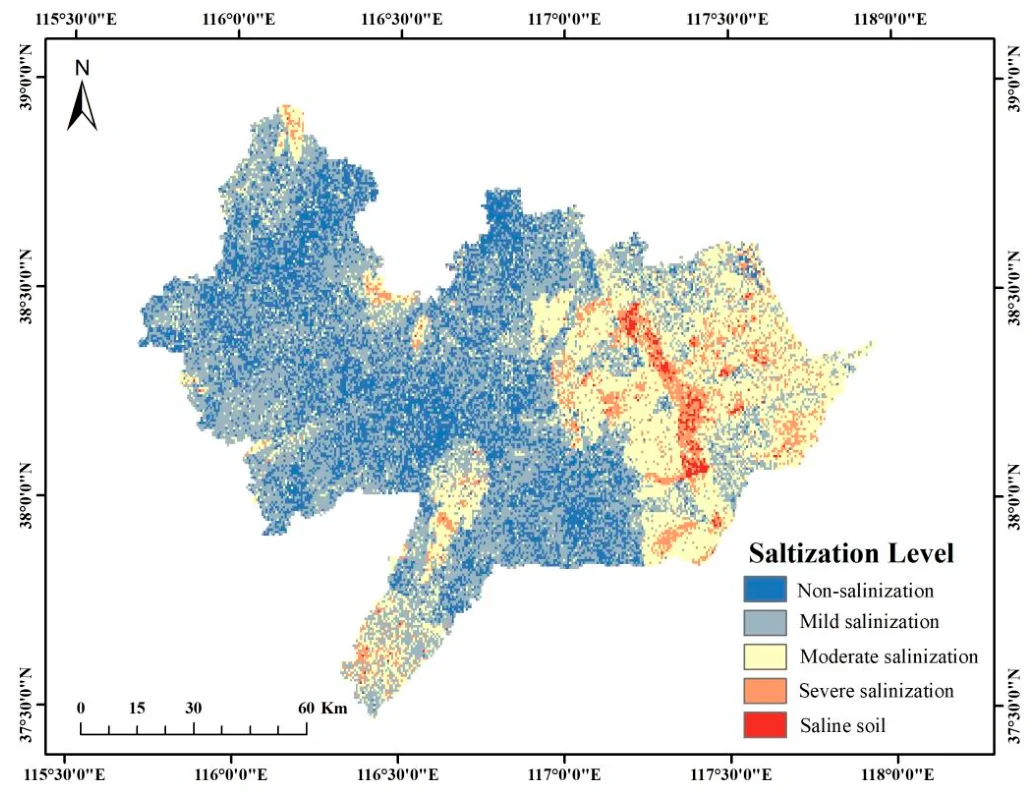
Spatial distribution map of soil salinity in the Cangzhou region derived from the CPO-CNN-SE-Attention model (with environmental variables included).

The trained CPO-CNN-SE-Attention model was applied to the wall-to-wall predictor layers generated from Sentinel-2-derived spectral indices and interpolated environmental variables to produce a continuous map of soil total salt content across the study area. The final prediction was based on eight spectral indices and the selected environmental variables identified through SHAP-based feature contribution analysis using the training dataset. The resulting soil salinity map is shown in Figure 9.

Figure 8 reveals pronounced spatial heterogeneity in soil salinity across Cangzhou City. Based on the predicted salinity classification, non-saline areas account for 25.13% of the study area, mildly saline areas for 41.93%, moderately saline areas for 25.72%, and severely saline plus saline soils for 7.40%.

The spatial distribution exhibits a distinct east-to-west decreasing gradient. Higher predicted salinity values were mainly concentrated in the eastern coastal region, which is consistent with previously reported coastal soil salinity patterns. These patterns may be associated with coastal environmental conditions and hydrological characteristics represented by the environmental covariates included in this study. Huanghua City and Haixing County showed relatively higher predicted salinity levels.

Away from the coastline, local geomorphological conditions introduce considerable spatial variability. Low-lying depressions, ancient river channels, and poorly drained areas corresponded with localized areas of higher predicted salinity. For example, higher predicted soil salinity was observed in Yaoqiao Town (Dongguang County), where local drainage conditions and soil properties may contribute to the observed spatial pattern.

Overall, the predicted spatial pattern indicates that soil salinity variability is associated with environmental gradients including coastal proximity, groundwater conditions, micro-topography, and soil properties within the studied region. The proposed framework effectively captures both regional salinity gradients and localized areas with relatively higher predicted salinity within the Cangzhou study area.

## 4. Discussion

Accurate spatial prediction of soil salinity requires representing the interactions among spectral responses, hydrological conditions, soil properties, and geographical factors. Models relying solely on spectral indices may be insufficient to fully represent the influence of hydrological, soil, and geographical conditions on soil salinity spatial variability. The performance of different machine learning approaches may vary depending on sample size, model structure, parameterization, and their ability to represent nonlinear interactions among heterogeneous predictors [47,48,49,50]. The results of this study indicate that integrating spectral and environmental information within a nonlinear prediction framework improves soil salinity spatial prediction performance compared with using spectral information alone and the evaluated benchmark models. The proposed framework achieved an RMSE of 0.928, an MAE of 0.720, and an R^2^ of 0.785, indicating improved predictive performance for regional soil salinity estimation. Rather than considering individual algorithms as independent contributions, the proposed framework integrates feature representation, feature weighting, hyperparameter optimization, and interpretation components within a unified prediction workflow. The CNN component provides nonlinear feature extraction from multisource inputs, while the SE attention mechanism adaptively recalibrates feature representations by emphasizing informative feature channels. The CPO optimization strategy enables automated hyperparameter selection and reduces dependence on manual adjustment. In addition, SHAP interpretation establishes a connection between model predictions and environmental variables, enabling the identification of variables with relatively higher contributions within the prediction framework. Therefore, the contribution of the proposed framework lies in the integration of prediction capability, feature representation, optimization efficiency, and model interpretability rather than the isolated application of individual algorithms.

The incorporation of environmental variables further enhanced model performance. Compared with the model using only Sentinel-2-derived spectral indices, the integration of environmental variables increased the coefficient of determination from 0.701 to 0.785, while reducing the RMSE from 1.084 to 0.928. These findings indicate that environmental variables provide additional predictive information beyond spectral information alone, particularly by representing variations in hydrological conditions, soil properties, and geographical characteristics. The KernelSHAP analysis further indicated that distance to the sea, pH, groundwater depth, surface structure, and silt content showed relatively higher contributions to model predictions within the study area. It should be noted that environmental variables may exhibit correlations with each other, which can influence the interpretation of SHAP contributions. For example, groundwater depth, soil moisture, soil texture, and distance to the sea may be spatially related in coastal environments. Therefore, SHAP values in this study should be interpreted as the contribution of each variable within the trained prediction framework rather than independent causal effects. The contribution of these environmental variables can be interpreted from the perspective of soil–water–salt processes. In coastal environments, shallow groundwater may enhance upward salt transport through capillary rise when evaporation exceeds precipitation and groundwater recharge, promoting salt accumulation in the near-surface soil layer. However, groundwater depth alone does not represent salt input directly, because groundwater salinity, soil hydraulic properties, and drainage conditions jointly regulate salt migration and accumulation. Distance to the sea may indirectly capture gradients in marine influence, groundwater conditions, and geomorphological characteristics associated with coastal salinity patterns. Soil texture variables, particularly silt content, influence soil water retention, infiltration, and drainage capacity, thereby affecting salt redistribution and retention processes. Soil pH may also reflect variations in soil chemical conditions associated with salt accumulation and soil development. Therefore, environmental variables provide complementary information describing the hydrological and soil processes controlling salinity variability, rather than simply acting as additional predictors. Future studies should further investigate variable interactions and multicollinearity effects using approaches such as correlation analysis, feature interaction analysis, or grouped interpretation methods.

The predicted spatial distribution is generally consistent with previously reported patterns of coastal soil salinity variability. Higher predicted salinity values were mainly concentrated in the eastern coastal area. This spatial pattern may be related to the combined influence of coastal proximity, groundwater conditions, evaporation intensity, and soil hydraulic characteristics represented by the environmental variables included in this study. In contrast, inland regions generally exhibit lower salinity levels, although localized salinity hotspots occur in poorly drained depressions and ancient river channels, potentially reflecting variations in topographic and hydrological conditions. The spatial pattern should not be interpreted as a direct observation of salt accumulation by satellite imagery. Sentinel-2 spectral variables capture integrated surface responses influenced by soil background, moisture conditions, and vegetation characteristics, whereas environmental variables provide additional information related to hydrological and soil conditions. Therefore, the predicted salinity distribution represents the combined effect of spectral and environmental information within the trained model.

To further evaluate the generalization capability of the proposed framework under spatially independent conditions, a spatial block cross-validation strategy was additionally conducted. Considering the spatial distribution characteristics of sampling locations, Cangzhou City was divided into ten spatial blocks according to county-level administrative units. During the K-fold validation process, each spatial block was alternatively used as an independent testing set, while the remaining blocks were used for model training. Compared with the random training–testing partition, the spatial block cross-validation resulted in an RMSE of 0.945, an MAE of 0.747, and an R^2^ of 0.766, while the random partition achieved an RMSE of 0.928, an MAE of 0.720, and an R^2^ of 0.785. The slight decrease in model performance under spatially independent validation indicates the potential influence of spatial autocorrelation among neighboring samples; however, the relatively small performance reduction suggests that the proposed framework maintains stable predictive capability beyond random sample partitioning.

Furthermore, considering the imbalance among different soil salinity levels, especially the limited number of samples representing severe salinity and saline soil categories, the agreement between predicted soil salinity classes and measured classification categories was further evaluated. After converting continuous predictions into salinity classes, the proposed model correctly identified 141 of 151 non-saline samples, 59 of 68 mildly saline samples, 49 of 54 moderately saline samples, 11 of 13 severely saline samples, and 15 of 18 saline soil samples, corresponding to accuracies of 93.38%, 86.76%, 90.74%, 84.62%, and 83.33%, respectively. These results indicate that although the limited number of extreme salinity samples may introduce uncertainty in the prediction of highly saline conditions represented by the available samples, the proposed framework maintained relatively consistent performance across the entire salinity range.

Despite the encouraging results, several limitations should be acknowledged. First, some environmental variables used in this study were derived from field measurements and subsequently interpolated into continuous raster layers. Therefore, interpolation uncertainty should be considered when interpreting the contribution of environmental variables. Although environmental layers were resampled to 10 m to ensure consistency with Sentinel-2 data, this spatial resolution represents data alignment rather than the intrinsic measurement scale of each environmental variable. Owing to the relatively sparse sampling density and the relatively coarse spatial resolution of some auxiliary environmental datasets, the interpolated environmental variables may not fully capture fine-scale spatial heterogeneity. Consequently, local salinity variability may be smoothed during feature construction, thereby affecting the spatial accuracy of the final soil salinity map. Second, although the proposed model achieved satisfactory prediction accuracy under random training–testing partitioning, potential spatial autocorrelation among neighboring samples may still lead to optimistic estimates of model performance. Sentinel-2 spectral variables may represent different surface processes under bare-soil and vegetated conditions. In vegetated croplands, spectral responses may be influenced by canopy coverage, crop type, phenological stage, chlorophyll content, and plant responses to salinity stress. Therefore, the relationship between spectral indices and soil salinity in this study should be interpreted as an indirect association rather than direct retrieval of soil salt content. Finally, groundwater depth was used as a hydrological indicator in this study, but groundwater salinity or mineralization data were unavailable. Since salt accumulation through groundwater depends on groundwater salinity, evaporation intensity, soil hydraulic properties, and drainage conditions, groundwater depth alone cannot fully represent groundwater-derived salt flux. Future studies should incorporate groundwater EC/TDS measurements and dynamic groundwater information to better characterize soil–water–salt processes. Future work will also explore the integration of higher-resolution environmental datasets and dynamic variables, such as evapotranspiration, groundwater fluctuations, and multi-temporal remote sensing observations, to further improve soil salinity spatial prediction accuracy and high-resolution mapping performance.

## 5. Conclusions

This study developed a multisource framework for spatial prediction of near-surface soil salinity in Cangzhou City by integrating Sentinel-2-derived spectral indices with environmental covariates. The framework combines nonlinear prediction, model interpretation, and hyperparameter optimization to evaluate the complementary information provided by spectral and environmental variables under the environmental conditions represented by the May 2024 dataset.

KernelSHAP analysis identified five environmental variables with relatively high contributions to model predictions, including distance to the sea, pH, groundwater depth, surface structure, and silt content. Among these variables, distance to the sea exhibited the highest contribution value (0.624), indicating that coastal proximity provided important predictive information within the developed framework.

The proposed deep learning framework achieved the highest predictive performance among all evaluated models, with an RMSE of 0.928, an MAE of 0.720, and an R^2^ of 0.785. The results indicate that integrating nonlinear feature representation, attention-based feature weighting, and hyperparameter optimization contributed to improved predictive performance within the evaluated dataset.

The integration of environmental variables further enhanced soil salinity spatial prediction performance, increasing the R^2^ from 0.701 to 0.785 while reducing the RMSE from 1.084 to 0.928. These findings indicate that environmental covariates provide additional predictive information beyond Sentinel-2 spectral responses alone by representing hydrological, soil, and geographical conditions associated with soil salinity variability.

Although the proposed framework achieved satisfactory performance in the Cangzhou study area, its applicability to other regions requires further validation. Future work should further investigate model generalization using broader spatial validation strategies and incorporate higher-resolution environmental datasets and dynamic hydrological variables. Overall, the proposed framework provides an interpretable approach for regional near-surface soil salinity spatial prediction under heterogeneous coastal environmental conditions.

## Figures and Tables

**Table 1:** Sources and characteristics of environmental variables used in this study.

Variable	Category	Data Source	Acquisition Time	Measurement/Derivation Method	Unit	Sample Size	Native Spatial Resolution
Groundwater depth	Hydrological	Field measurement	May-24	Field measurement and spatial interpolation	m	306	Point
Elevation	Topography	SRTM DEM	2024	Digital elevation model	m	Raster	30 m
Soil moisture content	Soil property	Field measurement	May-24	Laboratory measurement	%	306	Point
Clay content	Soil texture	Field measurement	May-24	Laboratory particle-size analysis	%	306	Point
Silt content	Soil texture	Field measurement	May-24	Laboratory particle-size analysis	%	306	Point
Sand content	Soil texture	Field measurement	May-24	Laboratory particle-size analysis	%	306	Point
pH	Soil chemical property	Field measurement	May-24	Laboratory measurement	–	306	Point
Surface structure	Soil property	Field survey	May-24	Field observation	–	306	Point
Geomorphic type	Geomorphology	Field investigation and geomorphological interpretation	May-24	GIS classification	–	306	Point
Distance to the sea	Geographic	OpenStreetMap coastline	2024	Euclidean distance analysis	km	Raster	Vector
Distance to rivers	Geographic	HydroSHEDS river network	2024	Euclidean distance analysis	km	Raster	15 arc-seconds (~500 m)

**Table 2:** Sentinel-2-derived spectral indices used as predictive variables and their calculation formulas.

Spectral Index	Formula	Reference
Normalized Difference Vegetation Index (NDVI)	NDVI = (B8 − B4)/(B8 + B4)	[10]
Salinity Remote Sensing Index (SRSI)	SRSI = ((NDVI − 1)^2 + SI^2)^0.5	[37]
Salinity Index SI1	SI = (B2 × B4)^0.5	[38]
Salinity Index SI2	SI2 = (B3^2 + B4^2 + B8^2)^0.5	[39]
Salinity Index SI3	SI3 = (B3^2 + B4^2)^0.5	[39]
Salinity Index SI4	SI4 = B11/B8	[39]
Salinity Index SI5	SI5 = (B4 − B11)(B4 + B11)	[40]
Salinity Index SI6	SI6 = B2 × B4/B3	[41]

**Table 3:** Statistical characteristics of measured soil salinity samples.

Salinity Level	Classification Standard (g/kg)	Sample Size	Minimum (g/kg)	Maximum (g/kg)	Mean (g/kg)	Standard Deviation (g/kg)	Coefficient of Variation (%)
Non-saline	<1	151	0.141	0.996	0.712	0.16	22.8
Mildly saline	1–2	68	1	1.97	1.372	0.29	21.3
Moderately saline	2–4	54	2.04	3.95	2.866	0.54	18.9
Severely saline	4–6	13	4.07	5.73	4.808	0.46	9.95
Saline soil	≥6	18	6.09	20.9	10.863	4.45	40.9
All samples		304	0.141	20.9	2.01	2.68	133.33

**Table 4:** Parameter settings of the CNN-SE-Attention model.

Parameter	Value
CNN number of filters	64
Convolution kernel size	[8, 1]
Pooling method	Global average pooling
Optimization algorithm	Adam
Training epochs	500
Initial learning rate	0.01
Learning rate decay factor	0.5

**Table 5:** CPO optimization settings and hyperparameter search ranges.

Parameter	Search Range
Learning rate	0.0001–0.01
Number of convolutional filters	16–128
Search strategy	Dynamic switching between exploration and exploitation phases
Fitness function	Validation RMSE
Optimization objective	Minimize RMSE
Dropout rate	0.1–0.5
Number of fully connected neurons	32–256
Population size	20
Maximum iterations	50
Switching parameter (R)	0.8

## Data Availability

The data presented in this study are available on request from the corresponding author and with permission of the Langfang Integrated Natural Resources Survey Center, China Geological Survey. The data are not publicly available due to confidentiality requirements.

## References

[1] Rengasamy P. (2016). Soil Salinization. Oxford Research Encyclopedia of Environmental Science.

[2] Huang D., Guo Y., Chen S., Wang Z., Sun B., Niu Y., Dong S. (2025). Different straw lengths and burial modes affect the salt distribution in coastal saline soil and the regulation of salt stress resistance in tomato. Plant Soil.

[3] Gao H., Fu T., Tang S., Liu J. (2023). Effects of saline water irrigation on winter wheat and its safe utilization under a subsurface drainage system in coastal saline-alkali land of Hebei Province, China. Irrig. Sci..

[4] He J., Den Q., Ma X., Su X., Ma X. (2021). Soil salinization affected by hydrogeochemical processes of shallow groundwater in Cangzhou City, a coastal region in North China. Hydrol. Res..

[5] Liu W., Shi T., Zhao Z., Yang C. (2025). Mapping Coastal Soil Salinity and Vegetation Dynamics Using Sentinel-1 and Sentinel-2 Data Fusion With Machine Learning Techniques. IEEE J. Sel. Top. Appl. Earth Obs. Remote Sens..

[6] Allbed A., Kumar L., Aldakheel Y.Y. (2014). Assessing soil salinity using soil salinity and vegetation indices derived from IKONOS high-spatial resolution imageries: Applications in a date palm dominated region. Geoderma.

[7] Zhang T.T., Zeng S.L., Gao Y., Ouyang Z.T., Li B., Fang C.M., Zhao B. (2011). Using hyperspectral vegetation indices as a proxy to monitor soil salinity. Ecol. Indic..

[8] Fang C., Wang Z., Yang X., Li Y., Xu X., Guo B., Li Z. (2022). Machine Learning Inversion Model of Soil Salinity in the Yellow River Delta Based on Field Hyperspectral and UAV Multispectral Data. Smart Agric..

[9] Liu X., Bai Y., Chen Z., Zhang J., Zhu J., Ding B., Zhang C. (2023). Multispectral remote sensing inversion and seasonal difference in soil salinity of cotton field in typical oasis irrigation area. J. Agric. Resour. Environ..

[10] Chen H., Zhao G., Chen J., Wang R., Gao M. (2015). Remote sensing inversion of saline soil salinity based on modified vegetation index in estuary area of Yellow River. Trans. Chin. Soc. Agric. Eng..

[11] Cao X., Ding J., Ge X., Wang J. (2020). Estimation of soil electrical conductivity based on spectral index and machine learning algorithm. Acta Pedol. Sin..

[12] Chen H., Ma Y., Zhu A., Wang Z., Zhao G., Wei Y. (2021). Soil salinity inversion based on differentiated fusion of satellite image and ground spectra. Int. J. Appl. Earth Obs. Geoinf..

[13] Yin H., Chen C., He Y., Jia J., Chen Y., Du R., Xiang R., Zhang X., Zhang Z. (2023). Synergistic estimation of soil salinity based on Sentinel-1 image texture and Sentinel-2 salinity spectral indices. J. Appl. Remote Sens..

[14] Huang Q., Xu X., Lü L., Ren D., Ke J., Xiong Y., Huo Z., Huang G. (2018). Soil salinity distribution based on remote sensing and its effect on crop growth in Hetao Irrigation District. Trans. Chin. Soc. Agric. Eng..

[15] Zhou X., Zhang F., Liu C., Kung H.-T., Johnson V.C. (2021). Soil salinity inversion based on novel spectral index. Environ. Earth Sci..

[16] Celik M.F., Isik M.S., Yuzugullu O., Fajraoui N., Erten E. (2022). Soil Moisture Prediction from Remote Sensing Images Coupled with Climate, Soil Texture and Topography via Deep Learning. Remote Sens..

[17] Sahbeni G., Ngabire M., Musyimi P.K., Székely B. (2023). Challenges and Opportunities in Remote Sensing for Soil Salinization Mapping and Monitoring: A Review. Remote Sens..

[18] Wang N., Chen S., Huang J., Frappart F., Taghizadeh R., Zhang X., Wigneron J.-P., Xue J., Xiao Y., Peng J. (2024). Global Soil Salinity Estimation at 10 m Using Multi-Source Remote Sensing. J. Remote Sens..

[19] Bandak S., Movahedi-Naeini S.A., Mehri S., Lotfata A. (2024). A longitudinal analysis of soil salinity changes using remotely sensed imageries. Sci. Rep..

[20] Javed T., Zhang J., Li W., Zhang J., Li J., Li H., Wang Z. (2025). Unlocking Soil Salinity Prediction With Remote Sensing Indices and Environmental Insights. Land Degrad. Dev..

[21] Sarkar S.K., Rudra R.R., Sohan A.R., Das P.C., Ekram K.M.M., Talukdar S., Rahman A., Alam E., Islam M.D.K., Islam A.R.M.D.T. (2023). Coupling of machine learning and remote sensing for soil salinity mapping in coastal area of Bangladesh. Sci. Rep..

[22] Jia P., Zhang J., Liang Y., Zhang S., Jia K., Zhao X. (2024). The inversion of arid-coastal cultivated soil salinity using explainable machine learning and Sentinel-2. Ecol. Indic..

[23] Wang Z., Zhang F., Zhang X., Chan N.W., Kung H.T., Ariken M., Zhou X., Wang Y. (2021). Regional suitability prediction of soil salinization based on remote-sensing derivatives and optimal spectral index. Sci. Total Environ..

[24] Abd El-Hamid H.T., Alshehri F., El-Zeiny A.M., Nour-Eldin H. (2023). Remote sensing and statistical analyses for exploration and prediction of soil salinity in a vulnerable area to seawater intrusion. Mar. Pollut. Bull..

[25] Wei Q., Nurmemet I., Gao M., Xie B. (2022). Inversion of Soil Salinity Using Multisource Remote Sensing Data and Particle Swarm Machine Learning Models in Keriya Oasis, Northwestern China. Remote Sens..

[26] Charilogis V., Tsoulos I.G., Tzallas A.T. (2023). An Improved Parallel Particle Swarm Optimization. SN Comput. Sci..

[27] Dutta P., Pal S., Kumar A., Cengiz K. (2023). Introduction to Genetic Algorithm. Artificial Intelligence for Cognitive Modeling.

[28] Mirjalili S., Lewis A. (2016). The Whale Optimization Algorithm. Adv. Eng. Softw..

[29] Abdel-Basset M., Mohamed R., Abouhawwash M. (2024). Crested Porcupine Optimizer: A new nature-inspired metaheuristic. Knowl.-Based Syst..

[30] Wang X., Luo Z., Li Z., Zhao Q., Dai J. (2023). The impact of groundwater recharge on land subsidence: A case study from the Cangzhou test area, Hebei Province, China. Hydrogeol. J..

[31] Xu Z., Chen S., Nachshon U., Mao X. (2025). Modeling evaporation dynamics from soils with major chlorides and high salinity. J. Hydrol..

[32] Eswar D., Karuppusamy R., Chellamuthu S. (2021). Drivers of soil salinity and their correlation with climate change. Curr. Opin. Environ. Sustain..

[33] Salehin M., Chowdhury M.D.M.A., Clarke D., Mondal S., Nowreen S., Jahiruddin M., Haque A., Nicholls R.J., Hutton C.W., Adger W.N., Hanson S.E., Rahman M., Salehin M. (2018). Mechanisms and Drivers of Soil Salinity in Coastal Bangladesh. Ecosystem Services for Well-Being in Deltas.

[34] Wang X., Zhang H., Zhang Z., Zhang C., Zhang K., Pang H., Bell S.M., Li Y., Chen J. (2022). Reinforced Soil Salinization with Distance Along the River: A Case Study on the Yellow River. SSRN Electron. J..

[35] Kirby K., Ferguson S., Rennie C.D., Cousineau J., Nistor I. (2024). Identification of the best method for detecting surface water in Sentinel-2 multispectral satellite imagery. Remote Sens. Appl. Soc. Environ..

[36] Mohammadi B., Pilesjö P., Duan Z. (2023). The superiority of the Adjusted Normalized Difference Snow Index (ANDSI) for mapping glaciers using Sentinel-2 multispectral satellite imagery. GISci. Remote Sens..

[37] Alhammadi M., Glenn E. (2008). Detecting date palm trees health and vegetation greenness change on the eastern coast of the United Arab Emirates using SAVI. Int. J. Remote Sens..

[38] Ding J., Yu D. (2014). Monitoring and evaluating spatial variability of soil salinity in dry and wet seasons in the Werigan-Kuqa Oasis, China, using remote sensing and electromagnetic induction instruments. Geoderma.

[39] Nicolas H., Walter C., Douaoui A.E.K. (2006). Detecting salinity hazards within a semiarid context by means of combining soil and remote-sensing data. Geoderma.

[40] Bannari A., Guedon A., El-Harti A., Cherkaoui F., El-Ghmari A. (2008). Characterization of slightly and moderately saline and sodic soils in irrigated agricultural land using simulated data of advanced land imaging (EO-1) sensor. Commun. Soil Sci. Plant Anal..

[41] Abbas A., Khan S., Hussain N., Hanjra M.A., Akbar S. (2013). Characterizing soil salinity in irrigated agriculture using a remote sensing approach. Phys. Chem. Earth Parts A/B/C.

[42] Zhou W., Yan Z., Zhang L. (2024). A comparative study of 11 non-linear regression models highlighting autoencoder, DBN, and SVR, enhanced by SHAP importance analysis in soybean branching prediction. Sci. Rep..

[43] Alreshidi I.M., Bisandu D.B., Moulitsas I. (2023). Illuminating the Neural Landscape of Pilot Mental States: A Convolutional Neural Network Approach with Shapley Additive Explanations Interpretability. Sensors.

[44] Balasubramani J., Rajendran S., Zakariah M., Alnuaim A. (2025). Ensemble of Deep Learning with Crested Porcupine Optimizer Based Autism Spectrum Disorder Detection Using Facial Images. Comput. Mater. Contin..

[45] Liu Z., Tao M. ECPO: An enhanced Crested Porcupine Optimizer. Proceedings of the 2024 4th International Conference on Communication Technology and Information Technology (ICCTIT).

[46] Huang H., Ding Q., Zhang J., Zhou Y., Pan X., Jia K. (2026). Ground-based Hyperspectral Coupled Interpretable Integrated Machine Learning for Salinity and pH Inversion in Agricultural Soils. Huanjing Kexue.

[47] Zhang J., Ding J., Wang J., Zhang Z., Tan J., Ge X. (2024). Spatial heterogeneity response of soil salinization inversion cotton field expansion based on deep learning. Front. Plant Sci..

[48] Zhang M., Fan X., Gao P., Guo L., Huang X., Gao X., Pang J., Tan F. (2025). Monitoring Soil Salinity in Arid Areas of Northern Xinjiang Using Multi-Source Satellite Data: A Trusted Deep Learning Framework. Land.

[49] Garajeh M.K., Malakyar F., Weng Q., Feizizadeh B., Blaschke T., Lakes T. (2021). An automated deep learning convolutional neural network algorithm applied for soil salinity distribution mapping in Lake Urmia, Iran. Sci. Total Environ..

[50] Chen H., Wu J., Xu C. (2025). Optimization of multi-source remote sensing soil salinity estimation based on different salinization degrees. Remote Sens..

